# Tumor-Derived Myeloid Cell Chemoattractants and T Cell Exclusion in Pancreatic Cancer

**DOI:** 10.3389/fimmu.2020.605619

**Published:** 2020-11-10

**Authors:** Robert H. Vonderheide, Adham S. Bear

**Affiliations:** Abramson Cancer Center, Department of Medicine, Perelman School of Medicine, University of Pennsylvania, Philadelphia, PA, United States

**Keywords:** myeloid, pancreatic, chemoattractant molecules, T cells, macrophages

## Abstract

Like many tumor types, pancreatic ductal adenocarcinoma (PDAC) exhibits a rich network of tumor-derived cytokines and chemokines that drive recruitment of myeloid cells to the tumor microenvironment (TME). These cells, which include tumor-associated macrophages and myeloid derived suppressor cells, block the recruitment and priming of T cells, resulting in T cell exclusion within the TME. Genetic or pharmacologic disruption of this chemokine/cytokine network reliably converts the PDAC TME to a T cell-high phenotype and sensitizes tumors to immunotherapy across multiple preclinical models. Thus, neutralization of tumor-derived chemokines/cytokines or blockade of their respective receptors represents a potentially potent strategy to reverse myeloid immunosuppression in PDAC, enabling benefit from checkpoint inhibition not otherwise achievable in this disease. Inhibition of oncogenic pathways that drive tumor-intrinsic expression of chemoattractants may be similarly effective.

## Introduction

A cancer inflammatory reaction dominated by myeloid cells is characteristic of many tumors, especially oncogene-driven invasive adenocarcinomas such as pancreatic ductal adenocarcinoma (PDAC) (1). The immunosuppressive cellular network established by tumor-derived myeloid cell chemoattractants – and the prospect of targeting this network therapeutically – has been increasingly understood, although the initial landmark observations underlying this concept were made by Mantovani and colleagues more than 35 years ago (2).

Experimental scrutiny of genetic mouse models of PDAC has uncovered a network of tumor-derived chemoattractants that promote myeloid cell infiltration from the earliest stages of tumorigenesis, and these observations have been validated to an increasing extent in PDAC patients. Myeloid cells in the tumor microenvironment (TME) block endogenous anti-tumor T cell responses and thwart effective utilization of checkpoint inhibitors. Given multiple redundancies in the myeloid compartment, *in vivo* depletion of myeloid cells in the TME in a clinically relevant fashion has been challenging. Disruption of this chemokine/cytokine network or the respective receptors may be more tractable with neutralizing antibodies or small molecular inhibitors that experimentally lead to loss of myeloid inflammation in the TME. Such strategies have resulted in effector T cell TME infiltration and T cell-dependent tumor regressions that can be further augmented with immunotherapy. Widespread clinical application of this strategy would require precision profiling of tumors to identify personalized targetable chemokine/cytokines or receptors. A potentially more generalizable and therapeutically effective alternative would be the development of inhibitors of the oncogenes that drive the tumor expression of these chemoattractants, e.g., inhibitors of mutant Kras. This review outlines the role of myeloid chemoattractants in promoting T cell exclusion in PDAC to establish immune privilege as well as suggests opportunities to improve cancer immunotherapy in scenarios where single-agent checkpoint blockade fails.

## PDAC Resistance to Checkpoint Blockade

Despite increasing label indications across numerous cancer histologies, antibodies that block CTLA-4, PD-1, and PD-L1 are essentially ineffective in patients with advanced metastatic PDAC, linked mechanistically to a number of tumor-intrinsic and –extrinsic factors in the TME (**Table 1**) (3). Combinations of anti-CTLA-4 and anti-PD-L1 have been equally disappointing (4). One exception are the <1% of all PDAC patients with high microsatellite instability (5), who often can respond to PD-1 checkpoint blockade and for whom pembrolizumab is now FDA-approved. Clinical trials continue to test the combination of checkpoint blockade with chemotherapy in metastatic PDAC, but initial results of gemcitabine, nab-paclitaxel, and nivolumab are discouraging. Thus, PDAC represents one of the most refractory tumors to currently approved checkpoint therapies, a particularly disappointing situation given the dire unmet medical need for a cancer that kills more individuals now than breast cancer and is predicted to become the second-leading cause of cancer death by 2030 (6).

**Table 1:** Obstacles in pancreatic cancer limiting utility of checkpoint blockade.

Category	Factor
Tumor intrinsic	Relatively low tumor mutational burden and neo-epitopes Low tumor PD-L1 expression Low rate of MSI high tumors (<1%)
T cell response	Relatively low T cell infiltration in most tumors Minimal baseline T cell priming against the tumor
Stroma	Dense stroma limiting drug delivery to TME Large inhibitory myeloid cell population Inhibitory cancer-associated fibroblasts

Numerous preclinical studies predicted the poor clinical activity of checkpoint inhibitors in PDAC patients. In the “KPC” genetically engineered mouse model of PDAC (in which mutant Kras and p53 are targeted for expression in the pancreas, resulting in a high fidelity model of the disease), there is no anti-tumor response in spontaneous tumors to single (CTLA-4, PD-1, PD-L1) or combination (CTLA-4 plus PD-1/PD-L1) immune checkpoint blockade (7, 8). In subcutaneous or orthotopic implantable models using KPC-derived, syngeneic tumor cell lines, responses to checkpoint blockade are only rarely observed (7). Classically, KPC tumors exhibit poor T cell infiltration and very low tumor mutational burden (TMB) that translates into few if any neo-epitopes (9–11). In other pancreatic models, where the TMB is higher, responses to checkpoint inhibition are observed at somewhat greater rates (12). These latter observations in non-KPC PDAC tumor models provide a rationale for the clinical evaluation of checkpoint blockade in PDAC patients. Despite the majority of human PDAC having low to very low T cell infiltration and TMB, about 20% of patients do exhibit T cell infiltration and a relatively elevated TMB, although there is no correlation between high T cell infiltration and high TMB (or neo-epitope burden) (13). The objective response rates in PDAC patients with single or dual checkpoint inhibition is far less than 20%; thus, neither T cell infiltration nor TMB serve as adequate predictive biomarkers of response in PDAC. Furthermore, PD-L1 expression as determined by RNA sequencing of primary PDAC tumors is among the lowest for any of other well-described immune checkpoint molecules including CTLA-4, VISTA, TIM3, TIGIT, LAG3, ADORA2A, or IDO1 (13). Moreover, there is no difference in PD-L1 expression among T cell-infiltrated vs. non T cell-infiltrated human PDAC, in contrast to the other checkpoint molecules such as CTLA-4 for which expression is significantly higher in T cell-infiltrated tumors (13). These findings raise the hypothesis that PD-L1 does not serve as the critical immune checkpoint that drives immunosuppression in PDA, consistent with the observation that the addition of nivolumab to a promising cancer vaccine in advanced PDAC patients fails to improve overall survival (14).

## Strategies to Sensitize PDAC to Checkpoint Blockade

Two primary strategies have been explored to sensitize PDAC patients to checkpoint inhibition (3). The first strategy hypothesizes that PDAC patients exhibit deficient T cell priming and a T cell response must first be mobilized to achieve efficacy with checkpoint inhibition (15). Immune priming strategies explored in combination with immune checkpoint inhibition include chemotherapy or radiation to induce immunogenic tumor cell death or the use of a cancer vaccine. This approach is highly effective in the KPC and other PDAC mouse models (8, 16, 17). To date, in PDAC patients, the combination of chemotherapy or cancer vaccines with PD-1 has not shown synergy. In our experience using the KPC model, the addition of agonistic CD40 antibody, aimed at licensing dendritic cells to activate anti-tumor T cells, has been the single most potent method to sensitize tumor-bearing mice to PD-1, CTLA-4, or combination immune checkpoint blockade – as has been recently reviewed (18). An ongoing national, randomized study is currently evaluating chemotherapy with or without agonistic CD40 mAb, with or without nivolumab, in first-line metastatic PDAC patients, based on promising phase 1b results with chemotherapy and CD40 mAb in the same patient population (NCT03214250). Other immune agonists such as those against stimulator of interferon genes (STING) or toll-like receptors (TLRs) represent additional approaches, as recently reviewed (3).

The blockade of novel checkpoint molecules represent the second strategy to sensitize PDAC tumors to PD-1/PD-L1 inhibition. Many of these novel checkpoints are highly expressed in PDAC and as noted above, these molecules increase in expression in the TME of tumors with higher levels of infiltrating T cells (13). However, evidence for single-agent activity of antibodies targeting these novel checkpoints in PDAC to date been minimal or modest, although preclinical data with select inhibitors (e.g. VISTA) are compelling (19, 20). However, as a sobering reminder, no novel checkpoint inhibitors have been approved by the FDA in oncology since the initial approvals of anti-CTLA-4 and anti-PD-1 aside from variations of PD-1/PD-L1 and combinations with anti-CTLA-4. As a telling example, the novel checkpoint inhibitor epacadostat (an IDO1 inhibitor) failed in combination with pembrolizumab in a randomized study in patients with advanced melanoma (21) despite compelling preclinical data in mice.

## The Myeloid Checkpoint in PDAC

Leukocytes dominate the PDAC microenvironment and among these, myeloid cells are typically the most over-represented, contributing to the well-described picture of cancer inflammation and desmoplastic reaction in this disease (9). This phenotype is reproduced in the spontaneous KPC model, in which infiltration by macrophages and myeloid-derived suppressor cells (MDSC) is evident in neoplastic lesions even before tumor cell invasion (9). The immunosuppressive effect of MDSCs on T cells in particular is striking in the KPC model and can be demonstrated both *ex vivo* and *in vivo* (22, 23). In both the KPC model and human PDAC, there appears to be an inverse relationship between myeloid infiltration and effector T cell infiltration (13). Thus, the question remains - is there a causal relationship between myeloid infiltration and effector T cell paucity? In the KPC model, it has been difficult to discern pharmacologically if myeloid cells are obstructing T cell infiltration because methods to eliminate myeloid cells *in vivo* are at best incomplete and transient (8). An alternative approach is to activate myeloid cells and re-educate (rather than deplete) them away from tumor-promoting activities. Such activation can be accomplished with agonistic CD40 antibody in the KPC model (24). With chemotherapy or radiation therapy, CD40 antibody can produce T-cell dependent tumor regressions (8, 16). Agonism of myeloid cell CD11b also repolarizes tumor-associated macrophages (TAM), reduces intratumoral myeloid cells, and leads to anti-tumor immunity in concert with checkpoint inhibition (25).

Myeloid cells in the TME are highly heterogeneous, and novel techniques such as ultra-high multiplex flow cytometry and single cell sequencing have unearthed significant complexities (26–28) which presents challenges for nomenclature systems to capture (29). Current views of myeloid heterogeneity extend far beyond designations of M1 vs M2 (or N1 vs N2); however, understanding the range of functionalities of various myeloid cells in the TME as either anti-tumor vs pro-tumor has been helpful conceptually. The rich mechanisms and dynamics that regulate myeloid cells and mechanisms of immunosuppression have been reviewed elsewhere (28, 30, 31).

Further indication that myeloid cells represent a major immune checkpoint in PDAC comes from studies in which individual cancer cells from spontaneous KPC tumors were cloned, re-implanted in syngeneic hosts, and upon tumor outgrowth harvested and inspected for T cell and myeloid cell infiltration (32). Although T cell-high tumors are unusual among spontaneous KPC tumors, KPC clones upon re-implantation are frequently T cell-high in this experiment. Interestingly, T cell-high tumors feature unusually poor myeloid infiltration, reproducing the human phenotype. In mixing studies administering T cell-low and T cell-high KPC clones, the T cell-low phenotype is dominant as outgrowing tumors are T cell-low and myeloid-rich (32). T cell-high tumors themselves are strikingly sensitive to anti-PD-1 and anti-CTLA-4 checkpoint therapy, even in the absence of CD40 agonism or chemotherapy (32). On the contrary, T cell-low tumors are refractory to anti-PD-1/anti-CTLA-4 recapitulating observations in the treatment of spontaneous KPC tumors.

## Myeloid Chemoattractants in PDAC

**Figure 1:**
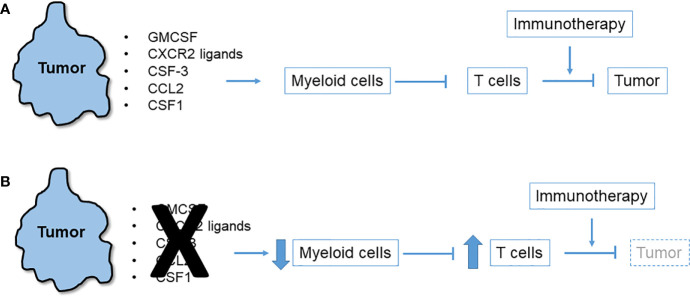
Immunosuppressive network of tumor-derived myeloid cell chemoattractants. **(A)** Multiple chemokines and cytokines released by pancreatic tumor cells trigger influx of myeloid cells to the tumor microenvironment (TME) that in turn suppress T cells that could otherwise attack the tumor or be induced to do so with immunotherapy. **(B)** Blockade or neutralization of tumor-derived chemoattractants in numerous mouse models leads to diminution of myeloid cells in the TME, an upsurge in infiltrating T cells, and tumor regression especially after immunotherapy.

Observations of T cell-high and T cell-low KPC clonal mixing studies led to the hypothesis that a local chemo-attractant factor elaborated by T cell-low tumor clones drives recruitment of myeloid cells to the TME. These myeloid cells in turn inhibit T cells and block their recruitment (**Figure 1**). In addition to the inhibition of T cell recruitment and effector function, myeloid cells may also produce factors that hinder dendritic cells in the TME, thereby preventing effective priming of anti-tumor effector T cell responses. For example, IL-6 has been shown to have such antagonistic effects on dendritic cells in PDAC (33). Consistent with this hypothesis, numerous studies have identified and characterized tumor-intrinsic pro-myeloid cytokines that regulate T cell immunosuppression in mouse models of PDAC. Examples include GM-CSF (22, 23), CXCR2 ligands (32, 34–36), CSF3 (32), CCR2 ligands (37), and CSF1 (38–40), as detailed below. Despite wide variation among cell lines as to which tumor-derived cytokine most prominently drives a myeloid-rich TME, precise ablation of a single, dominant cytokine for any given cell line reliably leads to T cell influx and immunotherapy responsiveness. In each case, the tumor cells themselves (albeit, not necessarily only the tumor cells) elaborate the cytokine or chemokine which are downstream products of either oncogenic mutant Kras or other driver pathways. Thus, oncogenic pathways can also enforce a myeloid-rich TME in addition to promoting oncogenic survival, proliferation and invasion (41). Rapid myeloid domination of the TME occurs from the earliest stages of tumor inception (9). Lineage tracing in the KPC model which permits the identification of metastatic tumor cells as isolated singlets in liver and lung identifies macrophages as accompanying lone tumor cells (42).

## GM-CSF

This growth factor is commonly expressed by tumor cells in both KPC mice and humans, even in early lesions (23). GM-CSF drives local proliferation of MDSC, which progressively accumulate in the PDAC microenvironment. When GM-CSF is genetically deleted from tumor cells in mice, T cell influx is triggered and tumors are rejected, but only in mice that are replete of CD8+ T cells (22, 23). GM-CSF is paradoxically understood to be a vaccine adjuvant and key component of promising pancreatic cancer vaccines (43), but as opposed to the low concentrations used in a subdermal vaccine, high concentrations of GM-CSF within the TME are immunosuppressive owing to effects on MDSC recruitment. In PDAC cells, GM-CSF production is downstream from mutant Kras signaling, linking immunosuppression to the driving oncogene (23).

## CXCR2 Ligands

CXCR2 ligands regulate myeloid trafficking into tumor cells. In humans with PDAC, high expression of CXCR2 is correlated with enrichment of intra-tumoral neutrophils (34). In the KPC model, CXCR2 blockade by genetic or pharmacologic means reduces recruitment of myeloid cells into the PDAC microenvironment (especially neutrophils), permitting T-cell dependent suppression of tumor growth, an effect which can be augmented by PD-1 inhibition to improve survival (34, 35). Although CXCR2 is highly expressed by cells in the tumor stroma, tumor expression of CXCR2 has also been observed in various genetic models and may drive autocrine or paracrine growth (44, 45). In KPC experiments, CXCL5 is the most prominent CXCR2 ligand produced by tumor cells, whereas stromal cells produce CXCL2 (34). Expression of tumor-derived CXCL5 is associated with mutant Kras expression and regulated by tumor NF-kB activation. In comparing T cell-high vs T cell-low KPC clones, CXCL1 – another CXCR2 ligand – is found to be the most differentially expressed (32). In these studies, CXCL2 and CXCL5, although known to be expressed in PDAC genetic models (45), are not differentially expressed. Genetic ablation of CXCL1 in T cell-low KPC clones abrogates the influx of myeloid cells in the TME, enabling infiltration by CD8+ T cells and rendering tumors universally responsive to anti-CTLA-4, anti-PD-1, and CD40 combination therapy (32). In contrast, overexpression of CXCL1 in T cell-high tumors reverses this phenotype and blunts response to immunotherapy. Epigenetic variations acting in concert with MYC in T cell-low vs T cell-high tumors underlie the differential regulation of CXCL1 (32). CXCL1 expression in the KPC system also depends on the necrosome, and in a study of KPC orthotopic tumors, RIP-1/RIP-3 driven necroptosis upregulates tumor-derived CXCL1 production and enhances peritumoral MDSC infiltration (36). RIP deletion reduces MDSC and triggers an influx of T cells and subsequent tumor regression. CXCL1 blockade similarly reduces MDSC in the tumors (36). RIP3 deletion is not, however, protective in B16 melanoma or subcutaneously implanted KPC tumors, indicating needed caution in explanatory models so as not to mistakenly imply that a single chemokine pathway is applicable across models or histologies.

## CSF3

Several studies also identify tumor-derived CSF3 (also known as G-CSF) as a cytokine that recruits myeloid cells to the TME, and CSF3 is associated with T cell inhibition and desensitization of PDAC tumors to immunotherapy (22, 32, 45).

## CCL2

Also known as MCP-1, CCL2 is a well-known tumor-derived macrophage chemoattractant in the TME (46, 47). Both human PDAC and KPC tumor cells express high levels of CCL2, although normal cells also express this chemokine (22, 37, 48). The elaboration of CCL2 in the TME results in mobilization of CCR2+ monocytes that are immunosuppressive. Although CCR2 inhibitors show promise in depleting TAMS *in vivo* in patients (37, 49), a compensatory mechanism of CXCR2+ neutrophils frustrates anti-tumor efficacy. The combination of both CCR2 and CXCR2 inhibitors in KPC mice prevents this compensatory reaction and results in significantly smaller tumors and improved survival, an effect that can be further enhanced with chemotherapy (50). As with other chemoattractants, the mechanism appears to be tumor-intrinsic. In KPC tumors, the epigenetic regulator HDAC5 inhibits Socs3, a negative regulator of CCL2, promoting CCL2 secretion and the recruitment of tumor-promoting macrophages to the TME (51).

## CSF1

Also known as M-CSF, CSF1 is commonly highly expressed by mutant Kras engineered mouse tumors (22, 38) and human PDAC cells. Inhibition of CSF1 or CSF1-R using blocking antibodies or small molecule inhibitors leads to the selective depletion of TAMs in the TME in pancreatic mouse tumor models (38, 39) and promotes tumor regression in combination with anti-PD-1 therapy with or without cancer vaccination (38, 40).

## Other myeloid cell regulators in the PDAC TME

Non-cytokine, tumor-intrinsic mechanisms of myeloid cell regulation are also active in PDAC mouse models. The tyrosine kinase EPHA2, for example, is markedly overexpressed in human and murine PDAC and functions to enforce a myeloid-rich, T cell-low phenotype in the TME (52). Knock-out of EPHA2 in tumor cells reverses T cell exclusion, dampens myeloid cell infiltration and sensitizes PDAC tumors to T cell-dependent rejection in response to immunotherapy. Interestingly, prostaglandin endoperoxide synthase 2, the gene encoding COX2, is downstream of EPHA2 and its deletion in KPC mice also reverses T cell exclusion and sensitizes tumors to immunotherapy (52). This genetic phenotype can be largely reproduced with COX2 inhibitors in concert with immunotherapy, revealing a potentially tractable clinically translatable strategy. Another key interaction in the pancreatic cancer TME is the interaction between cancer-associated fibroblasts (CAFs) and stellate cells and myeloid cells. CAFs mold the extracellular matrix (ECM) and provide survival and migration signals to cancer cells, and hinder drug delivery and potentially effector T cell infiltration (53, 54). CAFs themselves secrete cytokines and chemokines and can regulate the immune cell milieu, recruit immune-suppressive cells, and inhibit effector cells (55, 56). There is great phenotypic and functional heterogeneity in PDAC CAFs, including one subpopulation with antigen-presentation function (57). These findings provide new therapeutic opportunities, e.g., administration of vitamin D receptor ligand alters inflammation and fibrosis in pancreatitis and tumor stroma (58).

## Targeting Myeloid Chemoattractants for Immunotherapy

As illustrated above, tumor-intrinsic chemoattractants recruit myeloid cells into the PDAC TME, block T cell priming, infiltration and effector function, and enable tumor immune escape and growth (**Figure 1**). Importantly, this myeloid network is operative early in neoplastic development, often before invasion – a pathological stage well-characterized in the spontaneous KPC model. Importantly, the tumor promoting effects of myeloid cells are reversible as robust depletion of myeloid cells accomplished with an engineered toxin prevents both initiation and growth of mutant Kras-driven PDAC in mice (59).

Given T cell exclusion is an early and robust feature of PDAC, at least in the KPC model, the paradigm of immune editing is to be reconsidered because there is no or little Darwinian pressure from a T cell attack from which tumors must escape (11). Such a paradigm paradoxically implies tumors remain sensitive to T cells following progression from non-invasive to invasive cancer should the barrier of myeloid immunosuppression be removed (15). The lack of immune editing perhaps explains why PD-L1, upregulated in response to IFN-gamma from T cells, is expressed at low levels in the majority of PDAC tumors (13). In PDAC patients, unleashed T cell immunity may actually be quite powerful as evidenced by the correlation between a PD-L1^low^/CD8^high^ tumor sub-phenotype and positive prognosis in PDAC (60) as well as improved overall survival in the few resectable patients who naturally develop strong T cell immunity to PDAC compared to patients in whom T responses are minimal to absent (61).

How can the myeloid checkpoint be exploited to unleash anti-tumor T cells of clinical significance? One approach is to eliminate TAMs and other myeloid cells directly (50). In KPC mice, eight methodologies – ranging from clodronate to antibodies to combinations – fail to deeply or durably deplete TAMs (even when *systemic* myeloid cells are successfully depleted in some cases) (8). These disappointing results in mice include the use of antibodies to CSF-1 or CSF-1R. Initial efforts in patients with similar approaches are underway.

A second approach would be to use antibodies that neutralize the dominant cytokine driving myeloid cell accumulation in the TME or the receptor to which it binds. As noted above, this may vary model-to-model, or in the clinic, patient-by-patient – requiring precision immune profiling to select the optimal neutralizing antibody or antibodies. Anti-cytokine antibodies are increasingly among FDA approved drugs for inflammatory conditions. Among clinical-grade but still experimental antibodies, anti-GM-CSF or anti-CSF1-R antibodies would be logical and reasonable in PDAC patients whose tumors express these cytokines. However, because some cytokines also play other roles in *promoting* immunity including against pathogens (e.g., GM-CSF and CXCL1), cytokine neutralization represents a potential double-edged sword and must be carefully considered.

A third strategy would be to inhibit the cancer-promoting pathway or oncogene that is driving the cytokine or chemokine production, representing a more proximal and potentially more tumor-specific approach. One example would be to inhibit mutant Kras. Mutant Kras has long been an elusive oncologic target, but recently novel Kras inhibitors are providing new hope toward this possibility (62), although the applicability of KrasG12C inhibitors to PDAC patients is largely limited by the rare prevalence of this mutation in this patient population. Nevertheless, inhibition of mutant Kras or its downstream signaling pathways such PI3K may block cytokine production thereby decreasing myeloid cell accumulation in the TME enabling T cell infiltration and responsiveness to immunotherapy. It is also possible that such inhibition might, as an added benefit, also block PD-L1 expression on PDAC cells themselves (63).

There are of course other features of the crosstalk between tumor cells, myeloid cells, and other elements of the stroma that may be therapeutically targetable, as recently reviewed (28, 64, 65). These approaches have been discussed elsewhere and include blockade of CD47 (66) or FAK1 (67) on tumor cells, as well as PD-L1 (7) or TREM2 on TAMs (68). Of interest, TREM2 on TAMs is upregulated by GM-CSF and CSF-1 in certain models (69).

Finally, tumor-derived cytokines represent just one of a growing number of examples of tumor-intrinsic mechanisms of immune suppression. Examples are well-described elsewhere (70–72) and represent immune checkpoints beyond CTLA-4 and PD-1 just like the chemoattractants described here.

## Conclusions

Preclinical studies using mouse models of PDAC uncover a rich network of tumor-derived cytokines and chemokines that drive the recruitment of myeloid cells to the TME, including TAMs and MDSCs. These cells block the influx and priming of T cells, contributing to T cell exclusion. Genetic or pharmacologic disruption of this chemokine/cytokine network converts the TME to T cell-high and sensitizes tumors to immunotherapy. Thus, neutralization of such tumor-derived factors or their receptors – or potentially inhibiting the tumor-intrinsic pathways that drive their production – represents a strategy to address the “myeloid immune checkpoint” not only in PDAC but also potentially other tumor types. Efforts to test this hypothesis in patients remain nascent.

## Author Contributions

RV conceived the project, and both authors wrote and edited the manuscript. All authors contributed to the article and approved the submitted version.

## Funding

Support from NCI grants R01 CA217176, P01 CA210944, K12 CA076931, P30 CA016520, and the Parker Institute for Cancer Immunotherapy.

## Conflict of Interest

RV reports having received consulting fees or honoraria from Celldex, Lilly, Medimmune, and Verastem; and research funding from Apexigen, Fibrogen, Inovio, Janssen, and Lilly. He is an inventor on licensed patents relating to cancer cellular immunotherapy and receives royalties from Children’s Hospital Boston for a licensed research-only monoclonal antibody.

The remaining author declares that the research was conducted in the absence of any commercial or financial relationships that could be construed as a potential conflict of interest.
